# 3-Methyladenine ameliorates liver fibrosis through autophagy regulated by the NF-κB signaling pathways on hepatic stellate cell

**DOI:** 10.18632/oncotarget.22539

**Published:** 2017-11-20

**Authors:** Bingying Wang, Huan Yang, Yinyin Fan, Yong Yang, Wei Cao, Yanwei Jia, Ying Cao, Kangyun Sun, Zhi Pang, Hong Du

**Affiliations:** ^1^ Department of Clinical Laboratory, The Second Affiliated Hospital of Soochow University, Suzhou, Jiangsu 215004, P. R. China; ^2^ Department of Cardiology, The North District of Affiliated Suzhou Hospital, Nanjing Medical University, Suzhou, Jiangsu 215008, P. R. China; ^3^ Department of Gastroenterology, The North District of Affiliated Suzhou Hospital, Nanjing Medical University, Suzhou, Jiangsu 215008, P. R. China

**Keywords:** liver fibrosis, hepatic stellate cell, 3-Methyladenine, autophagy, nuclear factor-kappaB

## Abstract

3-Methyladenine (3-MA) is a selective type III phosphatidylinositol 3-kinase (PI3K) inhibitor and also blocks autophagosome formation. However, the effect of 3-MA in liver fibrosis has yet to be determined. Recent studies have demonstrated that autophagy is closely related to activation of hepatic stellate cells (HSC), a process critical in the pathogenesis of liver fibrosis. And the transcription factor nuclear factor-kappaB (NF-κB) is proved to play an important role in autophagy-induced signaling pathways. Thus, inhibition of autophagy regulated by NF-κB signaling pathway in HSCs is a potential therapeutic approach for attenuating liver fibrosis. Our studies proposed that 3-MA attenuates liver fibrosis induced by carbon tetrachloride (CCl4), and inhibit the expression of autophagy markers and transcriptional regulator NF-κB of hepatic stellate cell *in vivo*. The function of inhibition of autophagy in activation of human hepatic stellate cell line LX-2 was blocked by the inhibitor of NF-κB *in vitro*. Conclusively, 3-MA ameliorates liver fibrosis through inhibition of autophagy regulated by the NF-κB signaling pathways on hepatic stellate cell.

## INTRODUCTION

Liver fibrosis is the final pathological consequence of a wide spectrum of chronic liver diseases, while chronic and persistent fibrogenesis will lead to irreversible cirrhosis, hepatocellular carcinoma and ultimately liver failure [1]. Liver fibrosis is characterized by the excessive accumulation of an extracellular matrix (ECM) [2]. Hepatic stellate cells (HSCs) are the main source of ECM. Upon chronic liver injury, HSCs transform into myofibroblast-like cells which accompanied by increased proliferative capacity and secretion of ECM [3, 4]. The HSC activation is a critical event in hepatic fibrogenesis, therefore suppressing their activation is now considered as a potential strategy for antifibrotic therapies [5].

Autophagy is a catabolic intracellular pathway, targeting defective organelles and excessive components to the lysosomes for degradation [6, 7], usually activated by energy restriction, stress or inflammation [8]. Recent studies have demonstrated that autophagy is believed to closely correlate with activation of HSCs [9]. HSC was activated with autophagic flux increasing but was inhibited by autophagy inhibitor [10]. Lipids from intracellular lipid droplets released via autophagy, are thought to serve as energy sources for trans-differentiation of HSCs into myofibroblast-like cells in both mouse and human tissues [11, 12]. Therefore, suppression of autophagy in order to inhibit HSC activation may be a promising therapeutic target for the treatment of liver fibrosis.

The nuclear factor-kappaB (NF-κB) is a transcriptional regulator of genes involved in the immune and inflammatory responses [13, 14], which plays an important role in autophagy-induced signaling pathways [15, 16]. Several genes involved in autophagy, such as the genes encoding Beclin-1 and LC3-II, are regulated by NF-κB [17, 18]. Regulation of NF-κB activation affects autophagy-relevant gene expression, leading to the induction of autophagy [19].

3-Methyladenin is one of most commonly used as an autophagy inhibitor by blocking autophagosome formation via the inhibition of type III Phosphatidylinositol 3-kinases (PI-3K) [20, 21]. However, the effect of 3-MA in liver fibrosis has yet to be determined. The aim of this study was to determine whether 3-Methyladenine can ameliorate liver fibrosis induced by carbon tetrachloride (CCl_4_), and whether these effects involve autophagy inhibition regulated by NF-κB in HSCs. These experimental results will be helpful to determine the mechanism by which 3-Methyladenine inhibits the activation of hepatic stellate cell and its potential effects on liver fibrosis therapy.

## RESULTS

### 3-MA attenuates CCl_4_- induced liver fibrosis and inhibits activation of HSC

**Figure 1:**
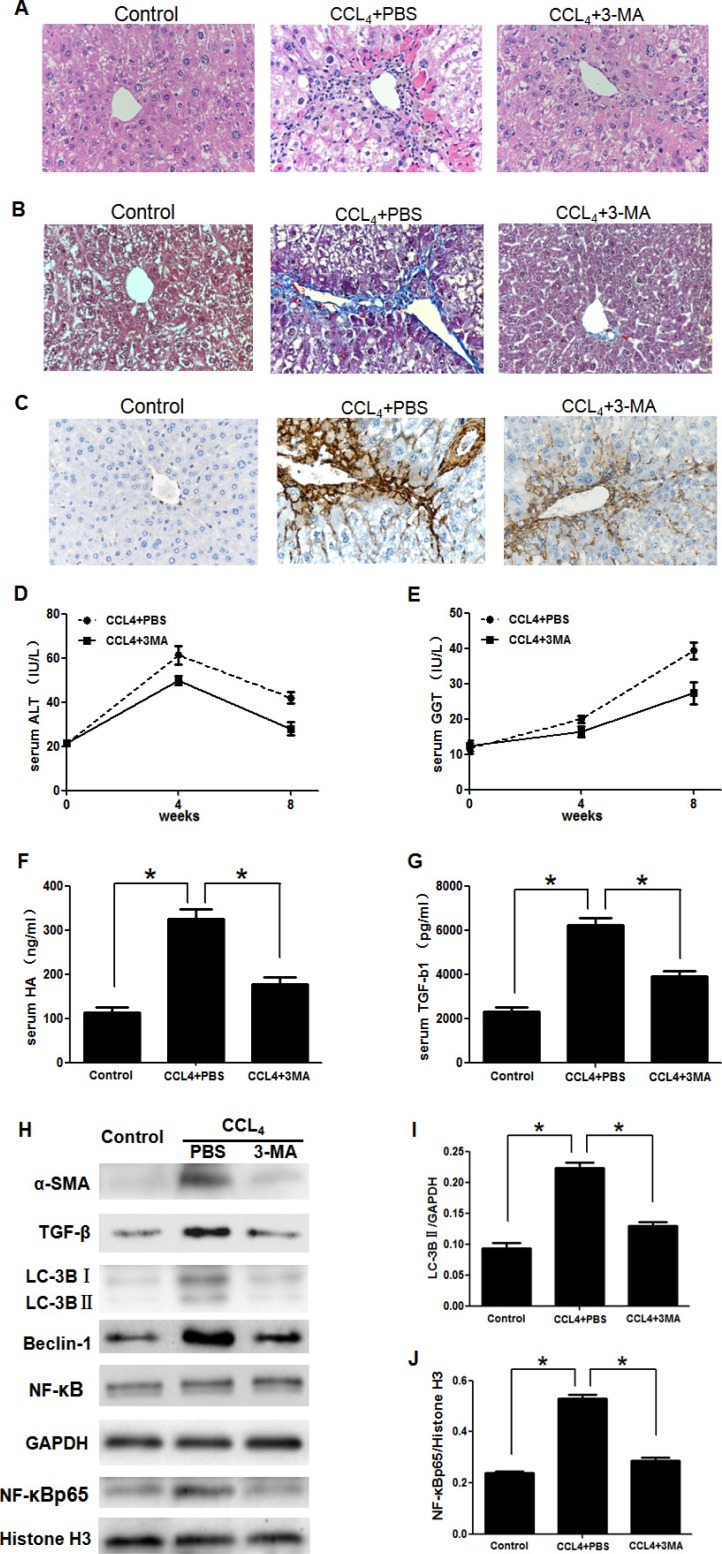
3-Methyladenine (3-MA) promoted the recovery of carbon tetrachloride (CCl4)-induced liver fibrosis by inhibiting the autophagy and translocation of NF-κB into the nucleus in HSCs (**A**) Hematoxylin and eosin dyeing of liver sections (200x). (**B**) Masson's trichrome stain of liver sections (200x). (**C**) Immunohistochemistry analysis of α-SMA(200x). (**D**, **E**) Serum alanine aminotransferase (ALT) and Glutamyl transpeptidase (GGT) levels. (**F**, **G**) Serum transforming growth factor-β1 (TGF-β1) and hyaluronic acid (HA) levels. ^*^P < 0.05. (**H**) Western blotting analysed expression of α-SMA, TGF-β, LC-3B, Beclin-1, NF-κB and NF-κBp65 in HSCs isolated from liver. (**I**, **J**) Density analysis of western blot bands. ^*^P < 0.05.

**Figure 2:**
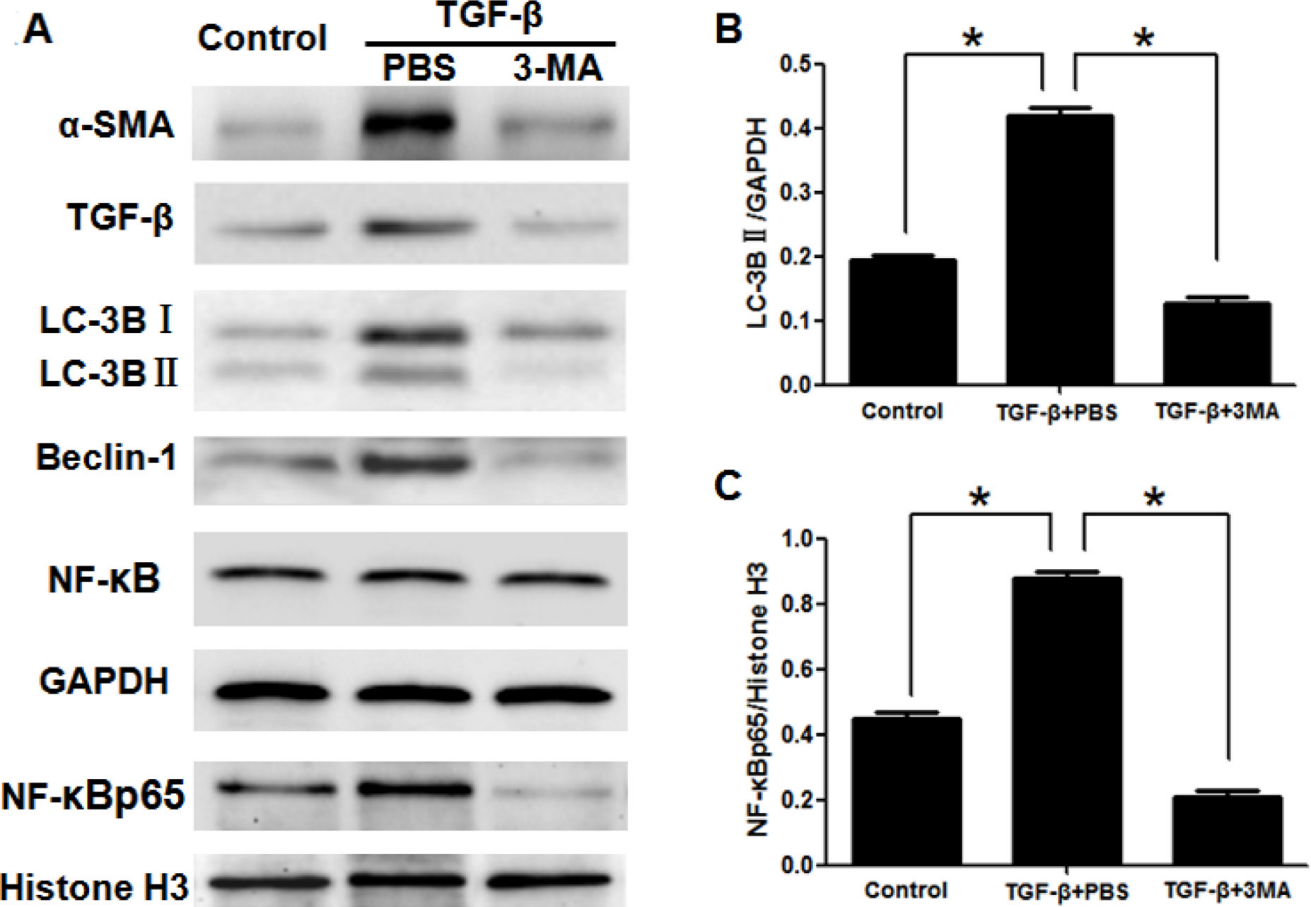
3-Methyladenine inhibited the activation of LX2 cells induced by TGF-β, and suppressed the autophagy and translocation of NF-κB into nucleus (**A**) Western blotting analysed expression of α-SMA, TGF-β, LC-3B, Beclin-1, NF-κB and NF-κBp65 in hepatic stellate cells. (**B**, **C**) Density analysis of western blot bands. ^*^P < 0.05.

To determine whether 3-MA could attenuate liver fibrosis induced by CCl_4_. Mice were divided into three groups described as method. Representative images of H&E staining showed that the number of destruction of hepatic lobules, apoptosis hepatocyte, and infiltration of inflammatory cells were significantly reduced in the CCl_4_+3-MA group, compared with CCl_4_+PBS group (Figure 1A). Blue/green matrixes indicated collagen deposition in tissue sections with MT dyeing. The normal liver has almost none of collagen deposition. Much of the collagen deposition was observed in the CCl_4_+PBS group livers, which decreased obviously after treating with 3-MA (Figure 1B). With the mouse treated by CCl_4_ for 8 weeks, the serum ALT, GGT, HA and TGF-β1 levels increased obviously in the CCl_4_+PBS group, while levels of CCl_4_+3-MA group almost kept normal (Figure 1D–1G). The expression of sign of activation of HSC α-SMA and TGF-β decreased obviously after treating with 3-MA *in vivo* (Figure 1C and 1H) and *in vitro* (Figure 2A).

### 3-MA inhibits activation of HSC in relation to autophagy

**Figure 3:**
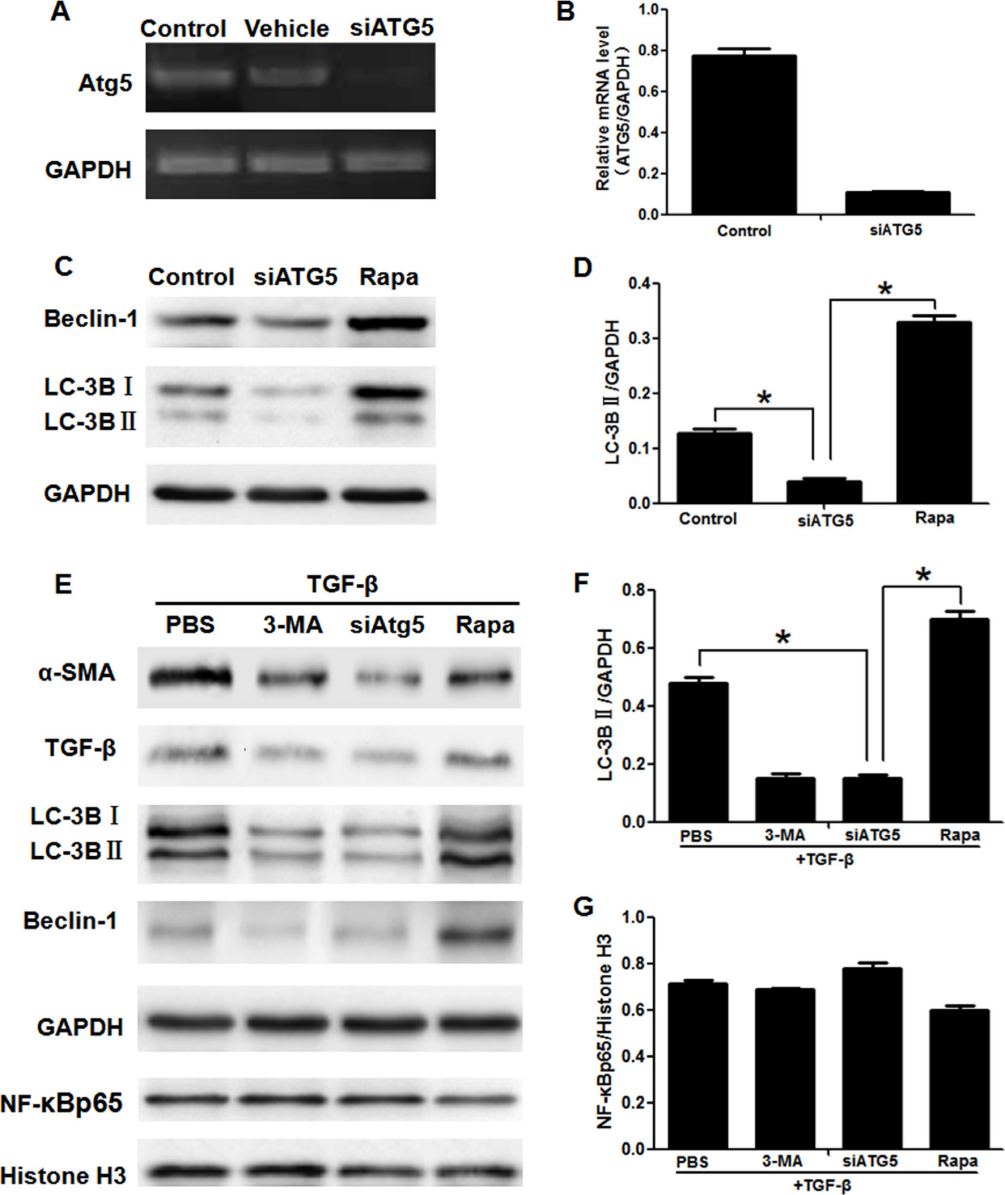
Blocking autophagy inhibited activation of LX2 cells, but having no effect on translocation of NF-κB into nucleus (**A**) The expression of mRNA ATG5 was assessed by RT-PCR in LX2 cells. (Control, LX2 cells were cultured with no compound; Vehicle, LX2 cells were cultured with ATG5 siRNAs in the absence of vehicle; siATG5, LX2 cells were cultured with siATG5 in the presence of vehicle). (**B**) Real-time PCR analyses of ATG5 mRNA levels in LX2 cells. (**C**) Western blotting analysed expression of LC-3B and Beclin-1 (Control, LX2 cells were cultured with no compound; siATG5, LX2 cells with siATG5 in the presence of vehicle; Rapa, LX2 cells were cultured with rapamycin). (**D**) Density analysis of western blot bands. ^*^P < 0.05. (**E**) Western blotting analysed expression of α-SMA, TGF-β, LC-3B, Beclin-1 and NF-κBp65 in LX2 cells. (**F**, **G**) Density analysis of western blot bands. ^*^P < 0.05.

Due to autophagy playing significant roles in the activation of HSC and 3-MA commonly used as an autophagy inhibitor [9], we detected whether the effect of 3-MA on inhibition of activation of HSC is related to autophagy. The expression of the autophagic marker protein LC3B of HSCs isolated from liver decreased obviously in the CCl_4_+3-MA group, compared with CCl_4_+PBS group (Figure 1H and 1I). *In vitro* model, the expression of protein LC3B and Beclin-1 of LX-2 cells also decreased in the TGF-β+3-MA group, compared with TGF-β+PBS group (Figure 2A and 2B). The 3-MA was confirmed to be able to inhibit autophagy of activated HSC. The expression of LX-2 cells activation markers α-SMA and TGF-β was suppressed by blocking autophagy with siATG5, but was accelerated by autophagic inducer rapamycin (Figure 3E). Therefore, the 3-MA inhibits activation of HSC in relation to autophagy.

### 3-MA inhibits translocation of NF-κB into nucleus

To determine the effect of 3-MA on translocation of NF-κB into nucleus, the nucleoprotein and plasma protein was extracted separately. NF-κB is a dimeric protein consisting of the Rel family. p50/p65 heterodimers, as the major Rel complex in the NF-κB family, modulate transcriptional activity in almost all cell types. The translocation of NF-κB into nucleus was displayed by expression of NF-κBp65 as an active form of NF-κB in the nucleus [22]. And Histone H3 was as an internal control of nucleoprotein. Although the expression of total NF-κB has no significant difference in HSCs and LX-2 cells (Figure 1H and Figure 2A), the translocation of NF-κB into nucleus was calculated by the ratio of NF-κBp65 and Histone H3 [23]. The NF-κBp65/Histone H3 increased in HSCs of CCl_4_+PBS group, which was inhibited by 3-MA (Figure 1H and 1J). The NF-κBp65/Histone H3 increased in LX-2 cells of TGF-β+PBS group, which was also inhibited by 3-MA (Figure 2A and 2C).

### The autophagy of HSC is regulated by NF-κB

**Figure 4:**
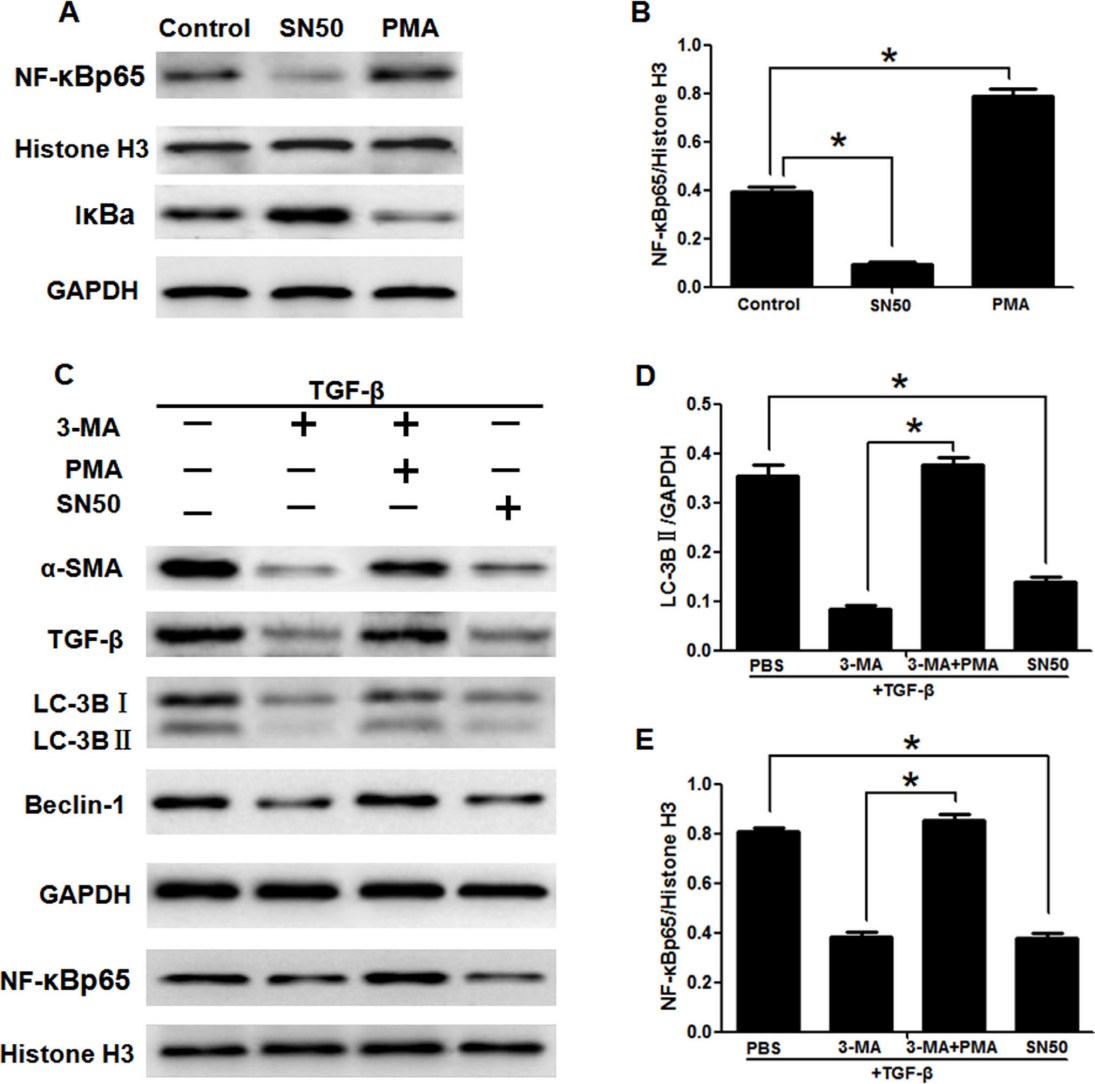
3-Methyladenine inhibited the activation of LX2 cells through autophagy regulated by the NF-κB signaling pathways (**A**) The translocation of NF-κB and its inhibitory protein IκBα of LX2 cells treated with SN50 or PMA were detected by western blotting. (**B**) Density analysis of western blot bands. ^*^P < 0.05. (**C**) The expression of α-SMA, TGF-β, LC-3B, Beclin-1 and NF-κBp65 in LX2 cells by western blotting. (**D**, **E**) Density analysis of western blot bands. ^*^P < 0.05.

In order to verify relationship between the autophagy and NF-κB in HSC, we blocked or promoted the expression of autophagy and NF-κB respectively. The expression of mRNA ATG5 of LX-2 cells was blocked by ATG5 siRNAs (Figure 3A and 3B). The expression of autophagic marker protein LC3B and Beclin-1 was also suppressed by siATG5 and induced by rapamycin (Figure 3C, 3D and 3F). As the effect of 3-MA, siATG5 inhibited the activation of LX-2 induced by TGF-β1 (Figure 3E). But siATG5 had no effect on translocation of NF-κB (Figure 3E and 3G). The specific inhibitor of NF-κB SN50 [24] suppressed the NF-κBp65/Histone H3 and increased the expression the NF-κB inhibitor IκBα (in the cytoplasm) [25], which effect was contrary to NF-κB inducer PMA [26] (Figure 4A and 4B). In addition to inhibiting the activation of LX-2 induced by TGF-β1 and expression of NF-κBp65/Histone H3 (Figure 4C and 4E), SN50 also inhibited autophagic marker protein LC3B and Beclin-1 (Figure 4C and 4D).

## DISCUSSION

Liver fibrosis is reversible in early stage of hepatic cirrhosis, while chronic and persistent fibrogenesis will lead to irreversible hepatocellular carcinoma or liver failure ultimately [27, 28]. Finding out an effective method to prevent the progression of fibrogenesis is an urgent problem for us. It is known that inhibition of HSC activation is an effective method to prevent the progression of liver fibrosis [5]. Therefore, studying the pathogenesis of HSC activation is helpful to discover new targets to inhibit fibrogenesis. Recent studies have revealed that autophagy is a newly recognized player in the activation of HSCs and subsequent formation of liver fibrosis [9]. And 3-MA recognized as a specific inhibitor of autophagy was proved to have an inhibitory effect on HSC activation [29, 30]. Whereas, the exact mechanism of this process is still rarely known, thus, further investigations are needed to clarify how 3-MA inhibits the activation of HSC.

In present study, first of all, we detected the effect of 3-MA on liver fibrosis *in vivo*. On the basis of the establishment of mouse liver fibrosis model, H&E staining and MT dyeing of liver tissue showed that 3-MA reduced inflammation, damage to hepatic lobular structure and collagen deposition and decreased the increasing serum ALT, GGT, HA and TGF-β1 levels induced by CCl_4_. As described above, we proved that 3-MA could ameliorate liver fibrosis. Due to the activation of HSCs plays an important role in hepatic fibrosis [5], we isolated HSC from the liver and detected the effect of 3-MA on HSCs. We found that 3-MA could reduce the expression of HSC activation markers α-SMA and TGF-β. In cell model, the expression of α-SMA and TGF-β of LX2 cells activated by TGF-β was also inhibited by 3-MA. Therefore, 3-MA ameliorates liver fibrosis is achieved by inhibiting the activation of HSCs.

Autophagy is a newly recognized player in the activation of HSCs and liver fibrosis progression [9], which supply energy for activation of HSCs by delivering triglycerides and other components [11, 12]. LC3II is located on the autophagosome membrane and is widely used as a marker to monitor autophagy [31]. Furthermore, the protein beclin-1 also participates in the process of autophagy [32]. Our results showed that expression of autophagy related proteins LC3B and Beclin-1 was reduced by 3-MA treatment both in HSCs from the primary culture of the liver and in LX2 cells, as compared to only treatment with CCl_4_ or TGF-β. In order to clarify whether this effect of 3-MA is related to autophagy, we blocked autophagy with siATG5. Atg5 is an essential autophagy regulator which involves in autophagosome generation [33]. Our results showed the activation of LX-2 cells was suppressed by blocking autophagy with siATG5, but was accelerated by autophagic inducer rapamycin. Therefore, the 3-MA inhibits activation of HSC in relation to autophagy.

NF-κB and autophagy are two major regulators involved in both pathological and physiological processes [34]. However, the association between these two signaling pathways still remains obscure. SN50 is an NF-κB cell permeable inhibitory peptide which contains the nuclear localization sequence of the transcription factor NF-κBp50 linked to a peptide cell-permeabilization sequence [24]. Our results showed that similar to the effect of the specific inhibitor of NF-κB SN50, the increasing translocation of NF-κB into nucleus induced by CCl_4_ or TGF-β1 was inhibited by 3-MA treatment both in HSCs from the primary culture of the liver and in LX2 cells. But this effect with 3-MA treatment was inhibited by NF-κB inducer PMA, which act as a transcriptional regulator. In the inactivated state, IκBα and NF-κB p65, p50 subunits in the inactivated state exists in the cytoplasm. The IκBα is degradated by activation of IKK (IκB kinase), resulting in activation of NF-κBp65 subunit and translocation from the cytoplasm to the nucleus. Thus, IκBα was used to reflect the translocation of NF-κB [25]. When the cells are stimulated by PMA, IkB dissociates from the trimer, exposing the translocation signal on the p50 subunit and the DNA binding site on the p65 subunit. The heterodimer exhibits NF-κB activity which is translocated from the cytoplasm into the nucleus and combinated with the NF-κB motif [26]. Therefore, PMA was used as inducer of NF-κB. In addition to inhibiting the expression of NF-κBp65/Histone H3, SN50 also inhibited autophagic marker protein LC3B and Beclin-1, which effect is opposite to PMA. However, both the specific inhibitor and inducer of autophagy had no effect on translocation of NF-κB. These results suggest that the autophagy of HSCs was regulated by the NF-κB pathways, nevertheless, autophagy showed little control over translocation of NF-κB.

Collectively, this research was the first to identify these potential anti-fibrotic mechanisms of 3-MA. We found that 3-MA treatment ameliorated CCl_4_-induced liver fibrosis via inhibition of autophagy regulated by the NF-κB signaling pathways on hepatic stellate cell. Together, these results indicate that 3-MA offer further insight into the development of drugs for the prevention and treatment of liver fibrosis.

## MATERIALS AND METHODS

### Animals

Mice (BALB/c, female, aged 6–7 weeks old) were purchased from the animal experimental center of Soochow University. All experimental procedures were in accordance with the Chinese legislation regarding experimental animals. Mice were randomly divided into three groups (*n* = 15) : Mice of CCl_4_+PBS group and CCl_4_+3-MA group received intraperitoneal injection with 20% volume fraction CCl_4_ (Nanjing Chemical Company) in olive oil at a dose of 2 mL/kg, control group were injected with the same amount of olive oil, while CCl_4_+3-MA group were injected additionally with 3-MA at a dose of 5 μg/g from the third week, twice per week. Caudal venous blood samples were collected per two weeks. The mice were sacrificed at 8 weeks post-infection of CCl_4_, and samples of liver and blood were collected.

### Histopathological staining

Liver tissues were processed for paraffin embedding and were sectioned into 4 μm sections. The sections were stained with hematoxylin and eosin and masson trichrome (MT) according to standard protocols. To analyze the extent of liver fibrosis, randomly picked fields of MT sections were captured from each animal.

### Immunohistochemistry

For immunohistochemical staining, the liver slides were incubated with diluted primary antibodies against anti-mouse α-SMA (1:500, abcam, USA), according to the manufacturer's instructions. The primary antibody was detected using the biotin-conjugated anti-rabbit immunoglobulin G antibody, and then incubated with streptavidin-biotin. After washing, the slides were colored with 3, 3-diaminobenzidine and counterstained with haematoxylin for microscopic examination. Removal of the primary antibody from the procedure provided a negative control.

### Serum assay

Serum Gamma-Glutamyl Transferase (GGT) and alanine aminotransferase (ALT) and transforming growth factor-β1 (TGF-β1) levels in mouse serum were detected by ELISA (mlbio, China). Hyaluronic acid (HA) levels in mouse serum were measured with the double antibody sandwich chemiluminescence method.

### Isolation and culture of HSC

BALB/c mice were treated according to the method described above. The HSCs were isolated from mice liver with the isolation kit (GENMED SCIENTIFIC, USA), according to the manufacturer's protocol. The cleaning solution was injected by the portal vein after anesthetizing mice and freeing the liver. After storing at room temperature for 5 minutes, the enzyme solution was injected. Strengthening solution also was injected by this way. The liver was surgically removed and chopped into small pieces with balance liquid, culturing at 37°C incubator for half an hour. The liver cell suspension is then filtered through a 100-μm cell strainer to eliminate undigested tissue remnants, and it is washed to remove excess digestion enzymes. The final step of the isolation procedure is a density gradient centrifugation. After resuspending the liver cell suspension with purification solution and centrifugal solution, centrifuging at 1450 g for 20 minutes. The top scattered white sample layer is HSCs. HSCs were cultured at 37°C in room air in incubator in culture medium with 5% CO_2_. Replace the culture medium every 48 hours until the cells are full of bottle.

### Cell culture, stimulation and chemical regents

Human hepatic stellate cell line, LX-2, was used as cell model. LX-2 cells seeded in 6-well plate were cultured at 37°C in room air in incubator in Dulbecco's modified Eagle medium (Gibco, USA) with 5% CO_2_ supplemented with 10% fetal bovine serum (Gibco, USA), 100 U/ml penicillin and 100 mg/ml streptomycin. LX-2 cells were randomly divided into groups: Control, LX-2 cells were cultured under normal condition; TGF-β+PBS, LX-2 cells were stimulated by 2 ng/ml TGF-β1 (Sigma, USA) for 72 hours; TGF-β+3MA, TGF-β+Rapa, TGF-β+SN50, TGF-β+PMA+3MA, after treating with TGF-β1 48 hours, LX-2 cells were cultured with 3MA (1 mg/ml, Sigma), rapamycin (200 μg/ml; Sigma, USA), SN50 (80 μg/ml), Phorbol 12-myristate 13-acetate (PMA, 30 ng/ml, Sigma, USA) and 3MA (1 mg/ml, Sigma, USA), respectively. Cells were collected after culturing 72 hours.

### Western blotting

Cells were collected harvested, pulverized and lysed in RIPA buffer. The nucleoprotein and plasma protein was separated by the nuclear and plasma protein isolation kit (Vazyme, USA). Equal amounts of protein were loaded and separated on SDS-PAGE gel. Proteins were transferred to polyvinylidene fluoride (PVDF) membranes (Millipore, USA). Sources and dilution factors of primary antibodies were: α-SMA (1:1000, abcam, USA), TGF-β (1:500, Bioworld, China), LC-3B (1:1000, abcam), Beclin-1 (1:1000, Proteintech), GAPDH (1:1000, Beyotime, China), NF-kB-P65 in the nucleus (1:500; SAB, China) and primary antibody nucleoprotein Histone H3 (1:1000; SAB). After incubation with the primary antibodies overnight at 4°C, membranes were incubated with HRP-conjugated goat anti-rabbit(1:1000, Beyotime, China), or goat anti-mouse antibodies (1:1000, Beyotime, China). This was followed by detection with luminata^TM^ crescendo western HRP substrate (Millipore, USA) and quantitated using MD Image Quant Software.

### RNA interference

Atg5 and negative control small interfering RNA (siRNA) were synthesized by Genepharma. The siRNA sequences were as follows: human Atg5 siRNA, sense: 5′-GAC GUU GGU AAC UGA CAA ATT-3′, and antisense: 5′-UUU GUC AGU UAC CAA CGU CTT-3′. The siRNA were transfected with Lipofectamine 2000 reagent (Invitrogen, USA) for 24 h in LX2 cells, according to the manufacturer's protocol.

### RNA extraction and quantitative real-time PCR

Total RNA was extracted with Trizol reagent (Invitrogen, USA) from LX-2 cells; cDNA was synthesized using a reverse transcription kit according to the manufacturer's instructions (Vazyme, USA). Quantitative real-time polymerase chain reaction (qRT-PCR) was used to detect the expression of ATG5 and β-actin genes. All samples were examined in triplicate, and all reactions were repeated three times independently using the CFX96 Touch™ Real-Time PCR Detection System (Bio-Rad).

### Statistical analysis

Data are expressed as the mean±standard deviation (SD). Statistical analysis was performed using Prism software (Graph Pad). Analysis of variance (ANOVA) and LSD-*t*-test were used to determine significant differences. Significant differences are indicated in the figure legends. All experiments were performed at least three times. Statistical ^*^P values < 0.05 were considered significant.

## Abbreviations

3-MA: 3-Methyladenine
HSC: hepatic stellate cells
NF-κB: transcription factor nuclear factor-kappaB
CCl_4_: carbon tetrachloride
